# The Antithrombotic Potential of Sulfated-Polysaccharides from Red Seaweed *Hypnea musciformis* (Wulfen) J.V. Lamouroux: An *In Vitro*, *In Silico* and *In Vivo* Study

**DOI:** 10.1021/acsomega.5c07355

**Published:** 2026-01-16

**Authors:** Caroline L. Peixoto, Vitória Karoline F. Monteiro, José Osmar S. Júnior, Lucas L. Bezerra, George Meredite C. de Castro, Norberto de Kássio V. Monteiro, Renato de Azevedo Moreira, Aline M. A. Martins, Ludmila Belayev, Reinaldo B. Oriá

**Affiliations:** † Laboratory of the Biology of Tissue Healing, Ontogeny and Nutrition, Department of Morphology and Institute of Biomedicine, School of Medicine, Federal University of Ceara, Fortaleza, CE 60020-181, Brazil; ‡ Neuroscience Center of Excellence, School of Medicine, 12258 Louisiana State University Health Sciences Center, New Orleans, Louisiana 70112, United States; § Department of Analytical Chemistry and Physical Chemistry, Federal University of Ceara, Fortaleza, CE 60020-181, Brazil; ∥ Department of Biochemistry and Molecular Biology, Federal University of Ceara, Fortaleza, CE 60020-181, Brazil; ⊥ Integrated Space Stem Cell Orbital Research (ISSCOR) Center/Sanford Consortium for Regenerative Medicine—UCSD, Center for Novel Therapeutics, 9310 Athena Cir, Suite 200, La Jolla, California 92037, United States

## Abstract

Thrombosis has emerged as a significant concern during the Coronavirus Disease 2019 (COVID-19) pandemic, with patients experiencing increased venous thromboembolism due to prolonged immobilization and inflammation. In Brazil, studies show a higher thrombosis risk among COVID-19 patients, emphasizing the need for effective thromboprophylaxis. Heparin (HEP), commonly used in hospitals, enhances antithrombin III (ATIII) activity to inhibit thrombin and factor Xa, thus reducing thrombosis risk. However, it can cause adverse effects like bleeding and HEP-induced thrombocytopenia, complicating its use and prompting the search for safer anticoagulant alternatives. This study aimed to evaluate the anticoagulant properties of sulfated polysaccharides (SP) derived from the red seaweed *Hypnea musciformis*, particularly their hydrolysates with different molecular weights. Additionally, computational analyses were conducted to investigate their interaction with ATIII, compared to HEP, to determine if the mechanism of action is similar. *In vitro*, the assays assessed the antithrombotic activity using activated partial thromboplastin time (APTT) and prothrombin time (PT) tests, with low-molecular-weight HEP CLEXANE (LMWH) as a positive control. Results showed that the intact polysaccharide and one hydrolysate (EX 5) prolonged activated partial thromboplastin time, while no samples affected prothrombin time. The *in vivo* bleeding time test revealed that these samples had a significantly lower hemorrhagic tendency than the positive control. Computational simulations indicated a stronger interaction between ATIII and the intact polysaccharide compared to its hydrolysate. These findings suggest that SP from *H. musciformis* could offer a promising anticoagulant therapy with reduced bleeding risk for clinical application in thrombotic conditions.

## Graphical abstract

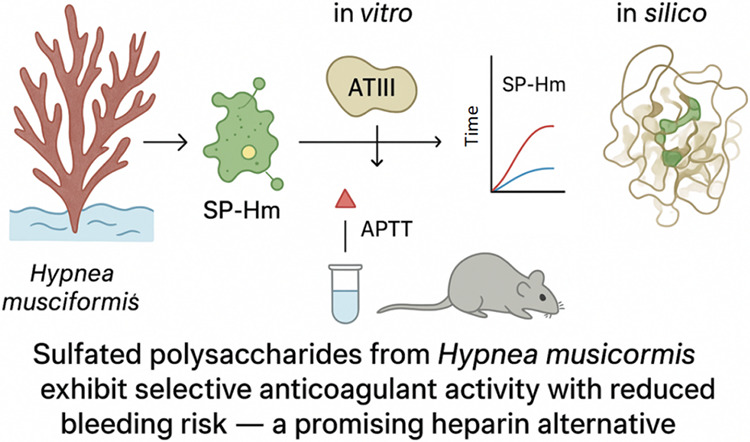

## 1 Introduction

Thrombosis is the formation of a clot or thrombus within a blood vessel and is one of the leading causes of death worldwide. A thrombus is an aggregate of platelets and fibrin accumulating in wounds to control bleeding and initiate healing. The body itself naturally breaks these thrombi. Sometimes, these thrombi can detach from the injury site, travel through the bloodstream, or form spontaneously in places without injury. It can cause occlusion of the vessels depending on their diameter, which is called thromboembolism. In more severe cases, the thrombus can occlude brain vessels, the so-called ischemic stroke, or pulmonary vessels, called pulmonary embolism, both of which can cause sudden death of patients.^1^

Antithrombotic drugs are classified according to their target in the thrombus formation process. Antiplatelet drugs (such as aspirin) inhibit platelet adhesion, aggregation, and release. Anticoagulants act on different molecules of the coagulation cascade to prevent the formation of the fibrin (insoluble) mesh. Fibrinolytic or thrombolytic agents convert plasminogen into plasmin, which degrades the fibrin mesh into fibrin degradation products, thereby dissolving the thrombus.^2^

Anticoagulant drugs such as unfractionated Heparins (UFH) and low-molecular-weight HEP CLEXANE (LMWH) are commonly used as prophylaxis to prevent thrombus formation.^3^ It is known that the mechanism of action of Heparin (HEPs) involves interaction with the natural anticoagulant antithrombin III (ATIII), which, when activated, inhibits both thrombin (factor IIa) and factor Xa, thereby blocking key steps of the coagulation cascade.^4^

LMWHs are derived from UFH by depolymerization, are more widely used because they offer pharmacokinetic and therapeutic advantages, such as a more predictable anticoagulant response, longer plasma half-life, greater bioavailability, convenience of administration, and reduction of side effects. However, the structural variability, production limitations, and safety issues of UFH and LMWH regarding animal origin are of concern. HEP has been confirmed to have adverse effects such as thrombocytopenia, arterial embolism, and hemorrhagic complications. Therefore, research is needed for new antithrombotic agents of nonanimal origin.^5,6^

It is important to note that a fully synthetic nonanimal anticoagulant, fondaparinux, is already available in clinical use. Fondaparinux is a chemically defined pentasaccharide that selectively potentiates ATIII-mediated inhibition of factor Xa, but it does not inhibit thrombin (factor IIa).^7,8^ In contrast, sulfated polysaccharides (SP) from *Hypnea musciformis* are heterogeneous, high–molecular weight galactans that can interact with ATIII in a broader manner, potentially influencing both factor Xa and thrombin inhibition depending on molecular weight and sulfation pattern.^9,10^ Furthermore, their structural diversity may provide complementary bioactivities such as anti-inflammatory, antiviral, and antioxidant effects.^10,11^ This distinction highlights the potential of marine polysaccharides as multifunctional alternatives, rather than direct analogues, to existing synthetic anticoagulants

It is recognized that SP from red seaweed have bioactive properties of great interest in the biomedical industry, among which are the antithrombotic and anticoagulant activities.^12^ However, the risk of bleeding is still a concern for SP safety. *H. musciformis* is a red macroalgae that contains kappa-carrageenan in its composition, which is an SP composed of alternating galactose and 3,6-anhydro-galactose, with one sulfate group per dimer. Kappa-carrageenan extracted from *H. musciformis* has demonstrated antimicrobial, anticancer, neuroprotective activities, and the ability to control inflammation in the colon.^13,14^ It is speculated that both antithrombotic activity and hemorrhagic tendency are associated with the molecular weight of the carbohydrate.^15^

The exploration of marine-derived compounds as anticoagulant agents has expanded significantly in recent years, motivated by the need for safer and more sustainable therapeutic alternatives. Red seaweeds, particularly those in the Rhodophyta phylum, are a rich source of SPs with distinct pharmacological properties, including anticoagulant, antiviral, antitumor, and anti-inflammatory effects.^16^ The structural diversity of SPs—governed by their sulfation degree, monosaccharide composition, glycosidic linkage, and molecular weight—plays a pivotal role in modulating their biological activity. Among the SPs, carrageenans stand out due to their presence in commercially and ecologically relevant red algae, such as *H. musciformis*. These molecules consist primarily of alternating α-1,3 and β-1,4-linked galactose residues, often substituted with sulfate groups, which confer negative charges critical for protein binding. Such interactions underpin their ability to affect coagulation pathways, particularly through binding to ATIII.^10^

In comparison to HEP, which are complex glycosaminoglycans derived from porcine or bovine tissues, SPs from marine algae offer multiple advantages. These include lower immunogenicity, absence of animal-borne pathogens, and the feasibility of large-scale cultivation under controlled conditions.^10^ Furthermore, marine SPs have demonstrated reduced side effects *in vivo*, such as minimized hemorrhagic risk, which is often a limitation in the clinical use of HEP.^11^

Previous investigations into SPs have emphasized the necessity of understanding structure–activity relationships, especially when considering antithrombotic function. It is now recognized that higher molecular weight polysaccharides may offer greater anticoagulant potency but could also increase the risk of adverse effects, including prolonged bleeding.^17^ Conversely, hydrolysates—low molecular weight derivatives—might retain biological activity with improved pharmacokinetics and diminished toxicity. In this context, hydrolysis becomes a valuable strategy not only for structure optimization but also for fine-tuning bioactivity. Hence, the present study integrates biochemical, *in vivo*, and *in silico* approaches to assess both the intact polysaccharides and their hydrolysates from *H. musciformis*, aiming to uncover the optimal molecular characteristics for safe and effective anticoagulant therapy.^17,18^

Due to the greater safety of LMWH compared to UFH in treating thrombosis and the prominent market for antithrombotic drugs, there is interest in finding alternative molecules for this purpose. The present study aimed to test the antithrombotic activity of sulfacted polysaccharide from *H. musciformis* (SP-Hm) and its hydrolysis products and their corresponding bleeding risk. In addition, we applied these *in silico* methodologies to compare the interaction of intact and hydrolyzed SP-Hm with ATIII, providing a mechanistic complement to our experimental observations. Molecular docking, molecular dynamics, and MM/PBSA simulations were applied to elucidate the mechanism of action of SP-Hm, comparing the interaction between SP-Hm with ATIII and UFH with ATIII. Collectively, these data contribute to the growing body of evidence supporting marine-derived SPs as viable alternatives to HEP, with the potential to advance anticoagulant therapy toward a more sustainable and safer clinical application.

## 2 Materials and Methods

### 2.1 Extraction of SP-Hm

The specimens of *H. musciformis* were collected from cultivation ropes at Flecheiras beach (03°13′06″ S–39°16′47″ W), Trairi, Ceará, Brazil, during the period from January to March. The seaweed was washed, and the epiphytes were removed and dried. Then the seaweed was ground and standardized for size. Subsequently, kappa-carrageenan was extracted according to the methodology described by Farias et al. (2000) with adaptations. Dried tissue (10 g) was suspended in 500 mL of 0.1 M sodium acetate buffer (pH 5,0) containing 5 mM EDTA, 5 mM cysteine, and 34 mL of papain 30 mg mL^–1^. The mixture was incubated at 60 °C for 6 h under constant mechanical agitation. After extraction, the system was filtered, and the supernatant precipitated with 10% cetylpyridinium chloride (CPC—Êxodo Cientifica, Sumaré—SP, BR) for 12 h at room temperature. After this time, the polysaccharides in the pellet were separated by centrifugation (5000*g*; 25 min; 25 °C), washed with 0.05% CPC and resuspended in NaCl-Ethanol solution (100:15 v/v), and then precipitated in ethanol for 24h. The new precipitate was filtered, washed 3 times with 80% ethanol, and dried with acetone. The extraction product was called SP-Hm.

#### 2.1.1 Quantification of Contaminant Protein Content

Bradford method^19^ was used to quantify protein content. A calibration curve was built using BSA to measure absorbance at 595 nm. A solution of 1 mg mL^–1^ of SP-Hm was used to quantify the protein content of the sample. 0.1 mL of 1 mg mL^–1^ SP-Hm and 2.5 mL of Bradford’s reagent were added to a test tube. After 10 min, the absorbance was read at 595 nm. The tests were performed in triplicate, and the concentration was estimated based on the readings obtained from the standard curve of BSA.

### 2.2 Animals

Wistar female rats (200–250 g) were obtained from the UNIFOR Central Vivarium and kept in the NUBEX Vivarium at 8 to 10 weeks of age. All animals received water and feed *ad libitum* with standard maintenance ration composed of crude protein (14–20%), ether extract (lipids) (3–6%), carbohydrates (40–60%), crude fiber (4–6%), minerals (4–8%) and moisture up to 12% and remained under controlled temperature conditions (22–25 °C) and light. All experimental protocols were performed according to the Guide to the Care and Use of Laboratory Animals (US Department of Health and Human Services) and approved by the Institutional Committee of Animal Care and Use of the University of Fortaleza (CEUA n° 2190151019/2019).

### 2.3 Hydrolysis

A central composite rotatable design (CCRD) of two independent variables and three levels with three central points was used to generate carbohydrates of different molecular weights and to evaluate the effects of hydrochloric acid concentration and temperature on the production of sulfated oligosaccharides from SP-Hm hydrolysis. The time and SP-Hm concentration was set at 30 min and 3%. The temperature ranged from 70 to 90 °C, and the hydrochloric acid concentration ranged from 1 mM to 300 mM, as shown in Table 1. Hydrolysis was performed in a 25 mL reactor with a water thermocirculator under mechanical stirring. After 30 min the hydrolysis was stopped by neutralizing the pH with sodium hydroxide. The hydrolysates were lyophilized for further analysis.

**Table 1:** Test Matrix with the Coded (and Real) Values of the Independent Variables, Three Central Points (C) of the Tests Carried out for the Acid Hydrolysis of PS-Hm from *H. musciformis* ^a^

	independent variables			reducing sugar (mg mL^–1^)	
EXP	[HCl] (mM)	*T* (°C)	molecular weight (kDa)	predicted	observed
**1**	–1 (44.8)	–1 (72.9)	20.70	0.0848	0.1294
**2**	–1 (44.8)	+1 (87.1)	7.23	0.3632	0.3410
**3**	+1 (256.2)	–1 (72.9)	1.52	0.4636	0.4197
**4**	+1 (256.2)	+1 (87.1)	1.56	0.4096	0.2987
**5**	–α (1.0)	0 (80.0)	231.00	0.0618	0.0322
**6**	+α (300.0)	0 (80.0)	1.75	0.3625	0.4582
**7**	0 (150.5)	-α (70.0)	8.86	0.3691	0.3549
**8**	0 (150.5)	+α (90.0)	1.72	0.5279	0.6083
**9 (C)**	0 (150.5)	0 (80.0)	2.76	0.5062	0.5542
**10 (C)**	0 (150.5)	0 (80.0)	6.51	0.5062	0.4857
**11 (C)**	0 (150.5)	0 (80.0)	7.56	0.5062	0.4787
**Intact**			263.00		

a Molecular weights were obtained by gel permeation chromatography (GPC) and Reducing Sugar contents (predicted and observed) by Dinitrosalicylic (DNS) colorimetric method.

### 2.4 Biochemical Characterization

#### 2.4.1 Molecular Weight Determination by Gel Permeation Chromatography (GPC)

The molecular weight of intact polysaccharides (SP-Hm) and oligosaccharides generated in the hydrolysis process was estimated by Gel Permeation Chromatography (GPC) according to the methodology of Mendes et al.^20^ Solutions of 0.1% of the hydrolysis products in ultrapure water were prepared, sonicated, and filtered on 0.45 μm-MILLIPORE membrane. GPC was conducted using a Shimadzu LC-20AD chromatograph (Kyoto, Japan) and a RID-10A refractive index detector. A 7.8 × 300 mm^2^ PolySep Linear (Torrance, USA) column with 0.1 M NaNO_3_ mobile phase at room temperature and 1.0 mL min^–1^ flow was used. The injection volume was 20 μL. The calibration curve for molar mass determination was constructed using pullulan standards (Shodex P-82, Showa Denko, Tokyo, Japan) with molar masses of 5.9 × 10^3^, 2,28 × 10^4^, 4,73 × 10^4^, 1,12 × 10^5^, 4,04 × 10^5^, 7,88 × 10^5^ and 2,28 × 10^6^ g/mol.

#### 2.4.2 Chemical Characterization by Infrared Spectroscopy

Infrared spectra were obtained with a Shimadzu Fourier-transform infrared spectrometer, model FTIR-8300, with a spectral region of 4000 to 400 cm^–1^. Potassium bromide (KBr) pellets were used for sample analysis.

#### 2.4.3 Quantification of Reducing Sugars by DNS

The quantification of reducing sugars (R.S.) by DNS is a colorimetric method. 3,5—dinitro salicylic acid (DNS reagent) reacts with the reducing sugar carbonyl carbon, reducing it to 3-amino-5-nitrosalicylic acid, a colored compound whose maximum light absorption occurs at 540 nm.^21^ The technique was applied according to dos Santos et al.,^22^ which used microplates to reduce reagent and sample consumption. The method was used to monitor hydrolysis and the increase in reducing sugars.

### 2.5 Antithrombotic and Anticoagulant Activities

Of the 11 experiments (hydrolysis), three were selected to be applied to the *in vitro* anticoagulant activity. The experiments EXP 1, EXP 5 and EXP 7 were selected based on molecular weight. LMWH enoxaparin (CLEXANE) was used as the positive control, and saline as the negative control.

#### 2.5.1 Activated Partial Thromboplastin Time (APTT)

The activated partial thromboplastin time (APTT) was performed following the manufacturer’s recommendations and analyzed in a coagulometer (CL2000B SINNOWA, Brazil). APTT measurements were performed using a kit obtained from BIOS Diagnóstica (CLOT APTT, Sorocaba—SP, BR). The assay was carried out in triplicate using LMWH enoxaparin (CLEXANE) as positive control and saline as negative control. Normal human plasma (90 μL) was mixed with 100 μL of APTT reagent 10 μL of EXP 1 (0.01–2 mg mL^–1^), EXP 5 (0.01–2 mg mL^–1^), EXP 7 (0.01–2 mg mL^–1^), Intact SP-Hm (0.01–2 mg mL^–1^), LMWH (0.01 and 0.1 μg mL^–1^) or saline. The samples were incubated at 37 °C for 3 min. To start the reaction, 100 μL of CaCl_2_ (0.025M) was added. The clotting time was recorded, and the results expressed in seconds.

#### 2.5.2 Prothrombin Time (PT)

The prothrombin time (PT) was performed according to the manufacturer’s recommendations and analyzed in a coagulometer (CL2000B SINNOWA, Brazil). The PT assay was performed using Thromborel S (Siemens, Munich, Germany),^23^ a lyophilized human placental thromboplastin reagent containing calcium chloride, stabilizers, and preservatives. According to the manufacturer’s instructions for use, this reagent does not contain any HEP neutralizer. In fact, the IFU specifies that normal plasma samples spiked with HEP above 0.6 U mL^–1^ yield abnormal results, confirming the absence of HEP-neutralizing activity. Therefore, the lack of effect observed in the PT assay reflects the selectivity of the SPs from *H. musciformis* for the intrinsic/common coagulation pathways, without interference from reagent composition. The assay was carried out in triplicate using LMWH enoxaparin (CLEXANE) as positive control and saline as negative control. Normal human plasma (90 μL) was mixed with 10 μL of EXP 1 (0.01–2 mg mL^–1^), EXP 5 (0.01–2 mg mL^–1^), EXP 7 (0.01–2 mg mL^–1^), Intact SP-Hm (0.01–2 mg mL^–1^), LMWH (0.01 and 0.1 μg/mL) or saline. The samples were incubated at 37 °C for 5 min. After the incubation, 200 μL of Thromborel S was added to start the reaction, and the clotting time was measured. The results were expressed in seconds.

### 2.6 Tail Transection Bleeding Time

The left jugular vein of rats was cannulated for injection of the samples. EXP 5 (2 mg kg^–1^), EXP 5 (1 mg kg^–1^), Intact SP-Hm (2 mg kg^–1^), and LMWH enoxaparin (CLEXANE) (0.3 mg kg^–1^) or saline were administered as a single injection. After 5 min, bleeding was induced by a section of the tail extremity 3 mm from the tip. The tails were blotted with tissue paper every 30 s and the time to cease bleeding was noted. For each treatment group (*n* = 6), the mean cessation of bleeding ± SD was determined

### 2.7 *In Silico* Analysis

#### 2.7.1 Semiempirical Calculations

The EX5 (Figure 1b) and intact SP-HM (Figure 1c) structures were drawn in Avogadro 1.1.1 ^24^ software and optimized using the Universal Force Field (UFF)^25^ with an initial energy minimization (2000 steps) *via* steepest descent algorithm,^26^ followed by a refinement with conjugate gradient algorithm^27^ (500 steps). Posteriorly, these structures were optimized again using the PM7 method^28^ in MOPAC 22.1.1 ^29^ software, these calculations were executed using the precise keyword to enhance convergence criteria.

**Figure 1:**
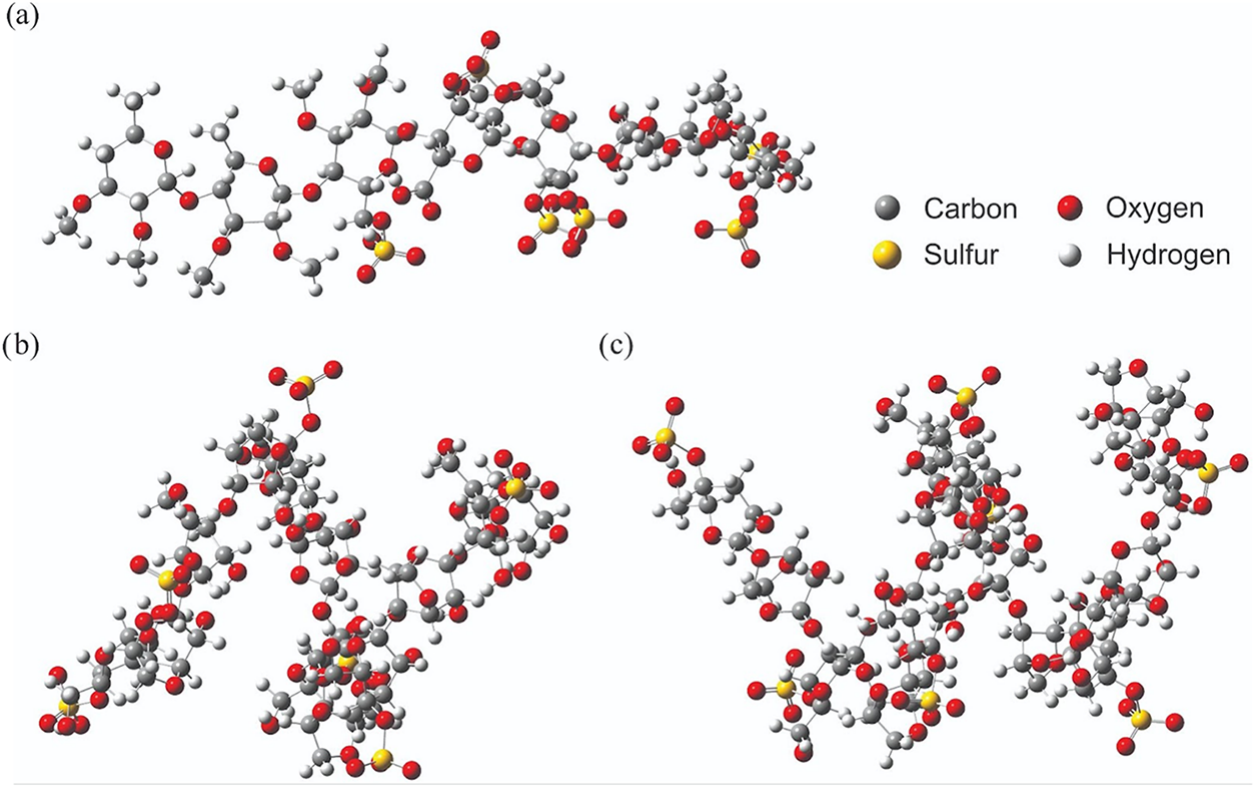
Optimized molecular structures used as input for molecular dynamics simulations. (a) HEP (reference compound), (b) SP-Hm hydrolysate fraction EX5, and (c) intact SP from *H. musciformis* (SP-Hm). The structures were geometry-optimized by Universal Force Field (UFF) prior to molecular dynamics simulations to ensure stable conformations for comparative interaction analysis with ATIII.

#### 2.7.2 Molecular Docking

The target used in this study was obtained from the RCSB Protein Data Bank (PDB: 1SR5)^30^ and prepared for docking by removing water and other hetero molecules from the original structure. Docking input files were created using AutoDock Tools^31^ 1.5.7 software. A three-dimensional box of dimensions 30 Å × 30 Å × 30 Å was created with central coordinates *x* = 22.893, *y* = −4.044, and *z* = 46.084, covering the entire ATIII active site region.^32^ Molecular docking was performed between ATIII and EX5, as well as ATIII and intact SP-HM, using Autodock Vina^33^ 1.5.7 software, obtaining nine best conformations for the ATIII-EX5 and ATIII-intact SP-HM complexes. The criteria used to choose the ligand conformation for molecular dynamics simulations are based on which conformation is closer to the HEP-binding domain.

#### 2.7.3 Molecular Dynamics

All the simulations were performed with the GROMACS 2023.2 (GROningen MAchine for Chemical Simulations) software.^34^ The EX5 and intact SP-HM protonation state chosen was based on physiological pH, as determined using Avogadro software. From this, the coordinate files were used as input to SwissParam,^35^ an external server in the CHARMM27 force field,^36^ which was used to parametrize the EX5 and intact SP-HM structures. The same methodology was also employed for the HEP molecule (Figure 1a) that was crystallized in the ATIII. The complexes were solvated in a dodecahedral box (9 Å × 9 Å × 16 Å) and neutralized by adding ions. The geometry of the systems was optimized using the steepest descent algorithm, followed by the conjugate gradient algorithm, both with 10^4^ steps with a tolerance energy of 10 kJ mol^–1^ nm^–1^. Posteriorly, the system was equilibrated with 20 ns NVT and 20 ns NPT ensembles at 1 bar pressure and 310.15 K temperature using the V-rescale thermostat^37^ and C-rescale barostat.^38^ Finally, a 100 ns production step was performed in three replicates with the Leap-Frog algorithm^39^ with the same pressure and temperature values as those from the equilibrium step.

The Interaction Potential Energy (IPE)^40^ analysis measures the strength of interaction between the ATIII and each molecule. This energy is obtained by the sum of the short-range van der Waals (*E*_vdW_) and electrostatic (*E*_ele_) energies, represented by eq 1. Besides, the *N*_*i*_ and *N*_*j*_ terms in this Equation are associated with the total number of atoms *i* and *j*, respectively.

1 $${\mathrm{IPE}}_{i,j}=\sum_{i}^{N_{i}}\sum_{j\neq i}^{N_{j}}E_{\mathrm{vdW}}\left(r_{\mathrm{ij}}\right)+E_{\mathrm{ele}}\left(r_{\mathrm{ij}}\right)$$

#### 2.7.4 MM/PBSA Simulations

The binding energy (Δ*G*_bind_) is used to estimate the spontaneous interaction between ATIII and the molecules, being calculated through eq 2. These calculations are based on the Molecular Mechanics Poisson–Boltzmann Surface Area (MM/PBSA)^41^ simulations using the g_mmpbsa tool.^42^ Only the last 10 ns of the production step from molecular dynamics were considered in MM/PBSA simulations.

2 $$\Delta G_{\mathrm{bind}}=\Delta E_{\mathrm{vdW}}+\Delta E_{\mathrm{ele}}+\Delta G_{\mathrm{polar}}+\Delta G_{\mathrm{non}‐\mathrm{polar}}-\mathrm{TS}$$

The Δ*E*_vdW_, Δ*E*_ele_, Δ*G*_polar_, and Δ*G*_nonpolar_ terms are associated with changes in van der Waals and electrostatic energies, and the polar and nonpolar contributions of the solvation Gibbs energy, respectively. Besides, the *T* and *S* terms in the same equation above are associated with the temperature (310.15 K) and the entropy, respectively.

### 2.8 Statistical Analysis

All the results are expressed as the mean ± standard deviation (S.D.). The elaboration of the central composite rotatable design (CCRD), the obtaining of predictive mathematical models, and the construction of the response surface methodology (RSM) (using pure error) were carried out using the Statistica 10.0 software (StatSoft, Inc.). For the statistical decision, “p” values less than 0.05 were considered significant. Statistical analyses were conducted using one-way Analysis of Variance (ANOVA), followed by Tukey’s post hoc test to identify significant differences between groups. A *p*-value <0.05 was considered statistically significant. Predictive mathematical models were developed considering the effects of factors on the studied response variables. The quality of the fit of the generated models was assessed using the *R* ^2^ determination coefficient.

## 3 Results

### 3.1 Extraction and Purity of SP-Hm

The average yield of extractions was 42.09 ± 5.91% in relation to dry seaweed. Carneiro et al. made the centesimal composition of *H. musciformis* and concluded that 54.24 ± 0.57% of the dry algae corresponds to carbohydrates.^43^ This difference between the extraction yield and the total carbohydrate is probably due to the cellulose content, which is not precipitated in this extraction method because it is not sulfated. Brito et al. obtained a yield of 31.8% using the same methodology and seaweed.^15^ In addition, no protein contaminants were detected using the Bradford method.

### 3.2 Molecular Weight Determination by Gel Permeation Chromatography (GPC)

The gel permeation chromatography method is suitable for estimating the molecular weight of SPs since it has high linearity, precision, and sensitivity.^44^ The molecular weight of the intact SP-Hm was 263 kDa, and the hydrolysates were between 1 and 231 kDa, as described in Table 1. Sulfated galactan compounds generally have a high molecular weight and a polydisperse profile, so the molecular weight was estimated based on the peak of the chromatography.^11,45^ EXP 1, 5, and 7 were selected according to their molecular weight (20, 231, and 8 kDa, respectively) to be tested for biological activities *in vitro*.

### 3.3 Chemical Characterization by Infrared Spectroscopy

The structure of the intact SP-Hm and the EXP 1, 5, and 7 were determined by FT-IR spectroscopy. The spectrum (Figure 2) exhibited peaks at 3462.22 cm^–1^ (O–H stretching), 2924.09 cm^–1^ (C–H stretching), 1629.85 cm^–1^ (bound water), 1267.23 cm^–1^ (O=S=O asymmetric stretching of ester sulfate), 1041.56 cm^–1^ (C–O–C stretching of 3,6-anhydrogalactose) and 848.68 cm^–1^ (C–O–S stretching of galactose-4-sulfate). Liu and co-workers^46^ obtained a similar spectrum from a commercial kappa-carrageenan, proving that the PS-Hm extracted was a kappa-carrageenan, and that the structure was maintained with hydrolysis, with only a decrease in the size of the polysaccharide.

**Figure 2:**
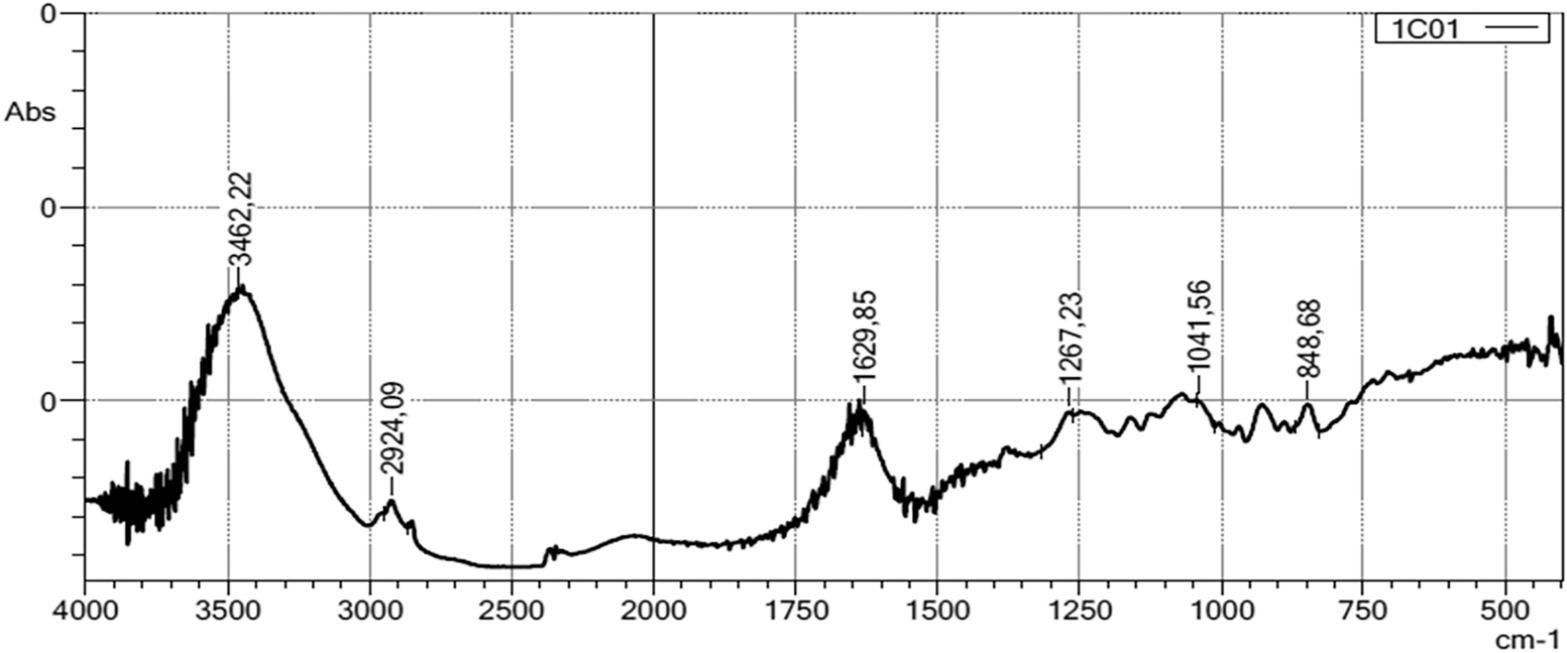
Fourier transform infrared (FT-IR) spectra of SP extracted from *H. musciformis* (SP-Hm). The absorption bands correspond to typical functional groups of SP, such as O–H stretching (∼3400 cm^–1^), C–H stretching (∼2930 cm^–1^), asymmetric S=O stretching (∼1250 cm^–1^), and C–O–S vibration (∼845 cm^–1^). These characteristic peaks confirm the presence of sulfate groups and the polysaccharide backbone.

### 3.4 Quantification of Reducing Sugars by DNS

The DNS methodology recognizes reducing sugar (RS) ends. During hydrolysis, as the polysaccharide is depolymerized, new reducing ends are generated; therefore, the higher the RS content, the smaller the polysaccharide or oligosaccharide generated. For this reason, the quantification of reducing sugars was adequate to accompany the hydrolysis process.

Table 1 also shows the contents of reducing sugars predicted and observed in the hydrolysates of the SP-Hm of *H. musciformis* obtained under different reaction conditions according to the CCRD. The observed results were close to those predicted, indicating that the execution errors were reduced. The highest concentration of reducing sugar was 0.60 mg mL^–1^, obtained in experiment 8, with a temperature of 90 °C and 150.5 mM HCl, At the same time, the lowest was 0.03 mg mL^–1^ in experiment 5 under the condition 80 °C and 1 mM HCl. These results show the relationship between the severity of hydrolysis and the concentration of RS generated. The mean and standard deviation of the RS average obtained from the three assays related to the central point was 0.50 ± 0.041 mg mL^–1^, suggesting the experiment’s reproducibility. The effects of temperature and acid concentration for the generation of RS by the acid hydrolysis process of the SP-Hm of the *H. musciformis* macroalgae are shown in Figure 3a. According to the Pareto, linear acid concentration was the independent variable that positively affected RS generation the most. However, the concentration of quadratic acid has a negative effect on the generation of RS. The temperature had no significant effects.

**Figure 3:**
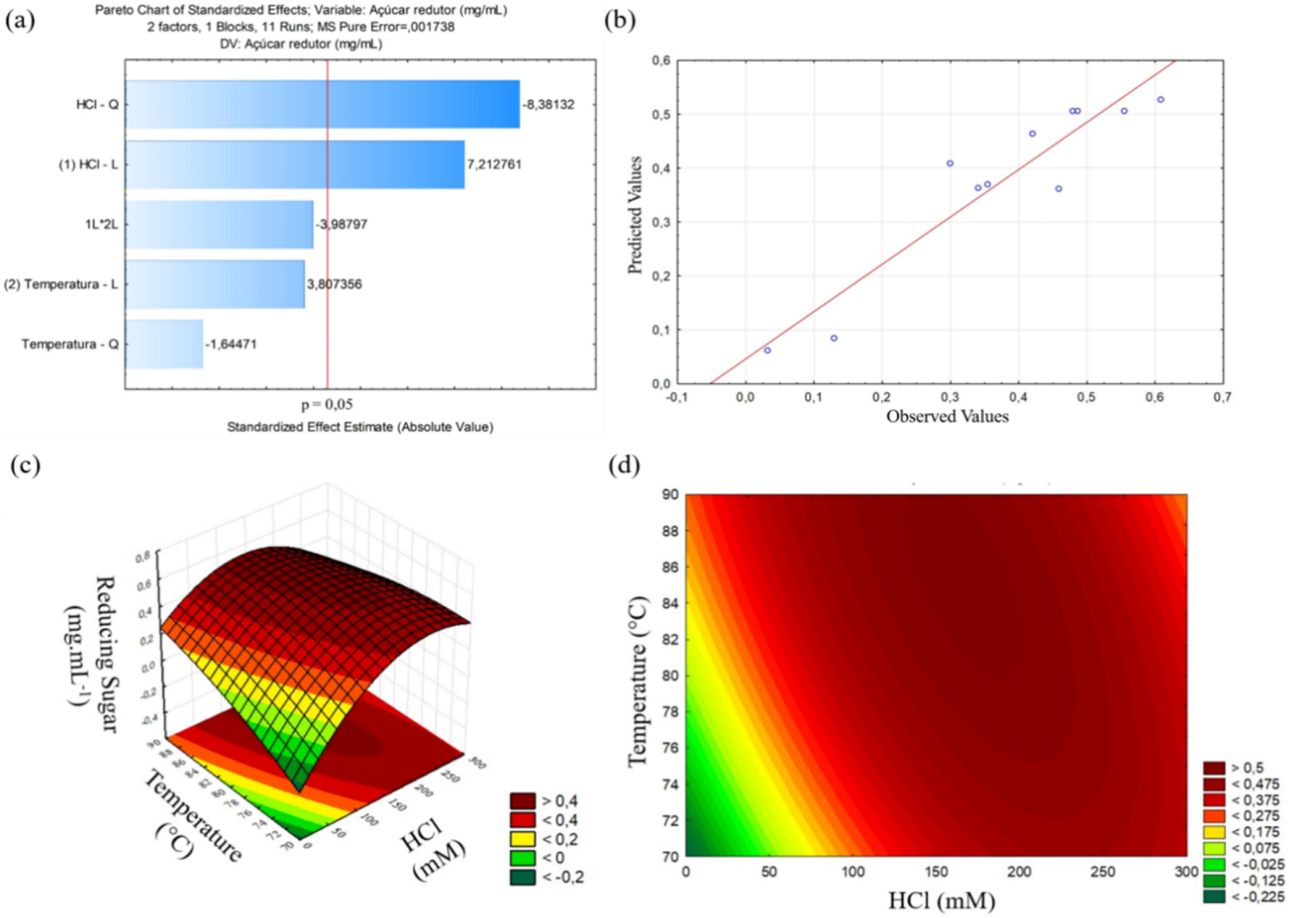
Optimization of reducing-sugar (RS) release from SP-Hm by acid hydrolysis. (a) Standardized Pareto chart showing the effects of temperature (°C) and hydrochloric acid (HCl, mM) on RS generation (α = 0.05). (b) Predicted *versus* observed RS values (mg mL^–1^) for the fitted response-surface model (*R* ^2^ = 0.87; solid line, *y* = *x*). (c) Response surface and (d) contour plot of RS as a function of temperature and HCl concentration; other factors were fixed (time = 30 min; SP-Hm = 3% w/v). Within the explored range (70–90 °C; 1–300 mM HCl), the model indicates a maximum near 85 °C and 166 mM HCl. RS was quantified by the 3,5-dinitrosalicylic acid (DNS) method.

The *F*_calculated_ values from the regression were *F*_Regression_ = 8.06 and *F*_Lackoffit_ = 5.75, the first being higher than the table and the second smaller, as shown in Figure 3b. The *F*_Regression_ value higher than the table indicates that the regression was significant and explains the results obtained based on the factors studied in the 95% confidence interval, and the lack of fit smaller than the table indicates that the model has a good fit. The coefficient of determination (*R* ^2^) value was 0.87, indicating a good fit of the model to the assessed response. This result was reinforced by the difference between *F*_calculated_ and *F*_tabulated_. The statistical significance of the model observed from the analysis of variance was confirmed by the normal distribution of the residuals presented between the experimental and theoretical values, shown in Table 2.

**Table 2:** Analysis of Variance (ANOVA) of the Mathematical Model for the Generation of Reducing Sugars by the Acid Hydrolysis of the SP-Hm of *H. musciformis* ^a^

source	sum of squares	degrees of freedom	mean sum of squares	*F*_cal_
regression	0.270040215	5	0.054008043	8.065117
residue	0.03348249	5	0.006696498	
lack of fit	0.030006518	3	0.010002173	5.755037
pure error	0.003476	2	0.001737986	
total	0.303523	10		
*R* ^2^	0.87			

a *F* (0.95; 5.0; 5.0) = 5.05; *F* (0.95; 3.0; 2.0) = 19.16.

The distribution shown in the graph shows that the values obtained in the experiments, represented by the circles, were close to the predicted values, represented by the line. The response surface methodology (RSM) defined the most appropriate conditions that maximize the generation of RS. The response surface graphs (Figure 3c) and contour curves (Figure 3d) for the response variable RS indicated the influence of temperature and acid concentration on hydrolysis. The critical values of the design for RS generation are 165.9 mM HCl and temperature of 85.4 °C. This means that values above or below these will lead to lower RS production.

### 3.5 Anticoagulant and Antithrombotic *In Vitro* Assays

#### 3.5.1 Activated Partial Thromboplastin Time

The action in intrinsic and common coagulation pathways was evaluated by the APTT test.^47^ The normal clotting time (negative control) in the test was 31.8s, and the clotting time with LMWH enoxaparin 0.1 mg mL^–1^ (CLEXANE, positive control) was 63.1s. The clotting time of each sample and each concentration is described on Figure 4a. Intact SP-Hm (265 kDa) and EX5 (231 kDa) in higher concentrations prolonged the normal clotting time (43.2 and 40.7, respectively). Liang et al.^48^ performed the APTT assay *in vitro* for commercial kappa-carrageenan (350 kDa) and a kappa-carrageenan oligosaccharide (3.4 kDa) and detected activity only in intact carbohydrates. For this reason, the EX5 and the intact SP-Hm were selected to assess the tail transection bleeding time *in vivo*.

**Figure 4:**
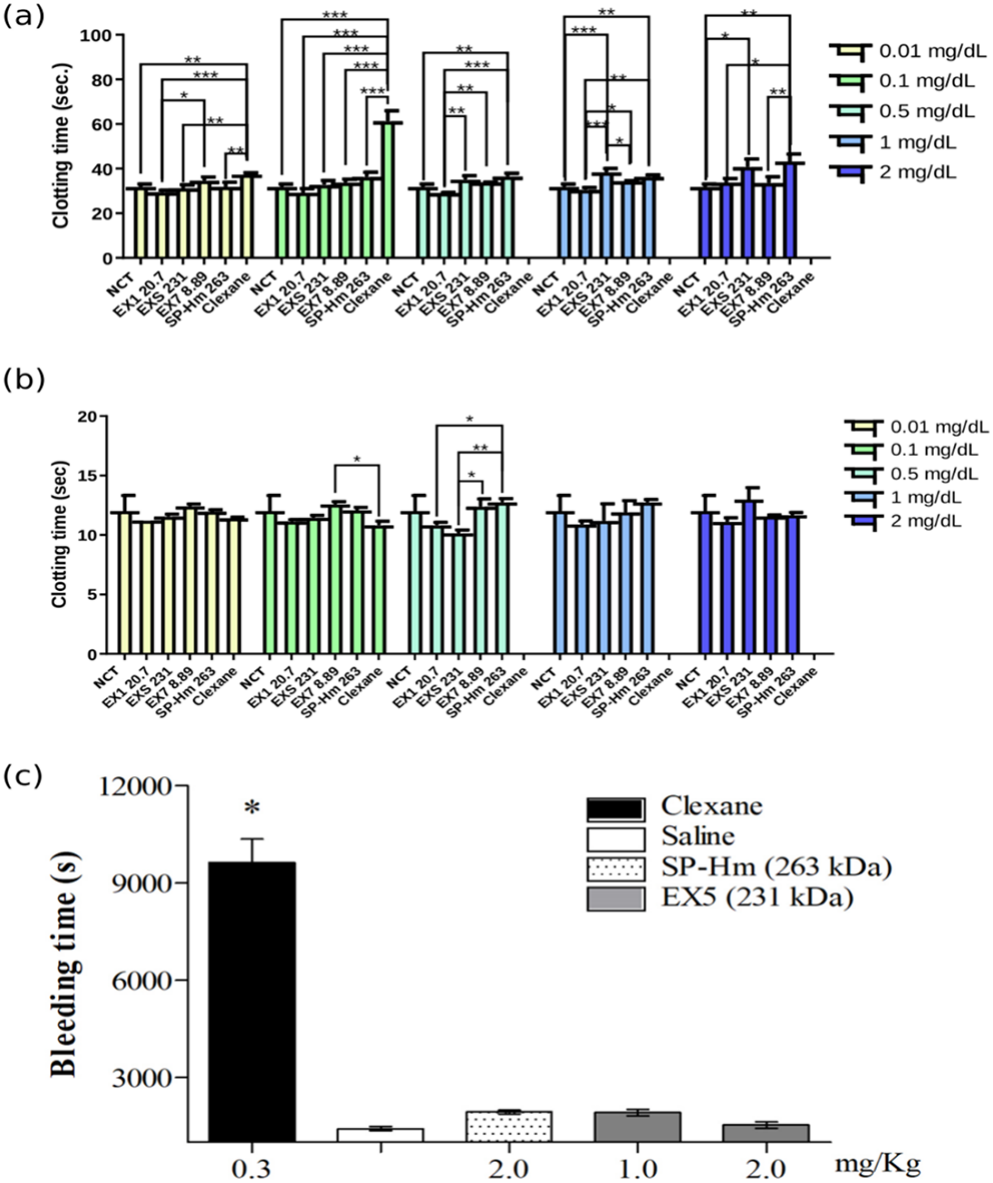
Anticoagulant activity of SP from *H. musciformis* and its hydrolysates. (a) Normal clotting time (NCT) compared with clotting times of SP-Hm hydrolysate fractions EX1, EX5, EX7, intact SP-Hm, and CLEXANE samples in the activated partial thromboplastin time (APTT) *in vitro* assay. (b) Normal clotting time (NCT) compared with clotting times of SP-Hm hydrolysate fractions EX1, EX5, EX7, intact SP-Hm, and CLEXANE samples in the prothrombin time (PT) *in vitro* assay. (c) Bleeding time in mice treated with intact SP-Hm, hydrolysate EX5, LMWH (enoxaparin, CLEXANE) as positive control, and saline as negative control. Data are expressed as mean ± standard deviation. Statistical significance was determined at *p* < 0.05.

#### 3.5.2 Prothrombin Time

The prothrombin time (PT) test addresses the extrinsic coagulation pathway, revealing deficiencies in the factors that are part of this system.^49^ The clotting times for each sample and concentration are presented in Figure 4b. None of the samples of different molecular weights and concentrations affected the PT test, showing no action on the extrinsic coagulation pathway.^50,51^ Therefore, SP-Hm acts selectively in the intrinsic coagulation pathway.

### 3.6 Tail Transection Bleeding Time

Antithrombotic drugs used clinically, such as HEP and its derivatives, have some serious side effects, including increased bleeding time, causing bleeding incidents.^52^ Therefore, it is crucial to assess the hemorrhagic tendency of potential antithrombotic drugs. The samples and concentrations with the best *in vitro* activity were selected to determine their hemorrhagic tendency. Therefore, the groups tested were EX5 1 mg kg^–1^, EX5 2 mg kg^–1^, SP-Hm intact 2 mg kg^–1^, and positive and negative controls. The bleeding time in the negative control group was 1416 s, which is considered normal. In contrast, the positive control, where LMWH enoxaparin (CLEXANE) was applied, had a bleeding time of 9633 s, 6.8-fold the normal bleeding time (Figure 4c). The samples analyzed, EX5 2 mg kg^–1^, EX5 1 mg kg^–1^, and SP-Hm mg kg^–1^, presented 1534, 1918, and 1936 s, respectively. No significant difference was observed between the samples and the buffer (negative control). Chagas and co-workers^53^ performed a bleeding time test with *Gelidiella acerosa* SP and obtained similar results at a dose of 1 mg kg^–1^, increased bleeding time by only 2.1 times (2627 s) when compared to the PBS control (1215 s).

### 3.7 *In Silico* Analysis

#### 3.7.1 Molecular Docking

Molecular docking simulations are computational methods frequently used to predict the best-fit conformation of a ligand on a protein.^54,55^ In this study, molecular docking analysis was performed between the ATIII target and EX5, as well as between the ATIII target and the intact SP-HM to determine the best conformation. These conformations will be utilized in molecular dynamics simulations. Table 3 shows that the ATIII-EX5 and ATIII-intact SP-HM complexes registered the affinity energy values of −6.76 and −4.94 kcal mol^–1^, respectively. Based on molecular docking results, both molecules exhibited a strong interaction with the ATIII target, especially the EX5 molecule.

**Table 3:** Affinity Energy Values of the ATIII-EX5 and ATIII-Intact SP *H. musciformis* Complexes from the Molecular Docking^a^

	EX5	intact SP-HM
conformation	Affinity (kcal mol^–1^)	Affinity (kcal mol^–1^)
**1**	–6.76	–5.52
**2**	–6.43	–5.47
**3**	–6.35	–5.24
**4**	–6.34	–5.10
**5**	–6.33	–4.98
**6**	–6.24	–4.94
**7**	–6.18	–4.79
**8**	–6.07	–4.37
**9**	–6.06	–4.01

a The values highlighted in red refer to the selected conformation for the molecular dynamics simulations.

#### 3.7.2 Molecular Dynamics

Molecular dynamics simulations are a widely used method for studying the stability of protein–ligand complexes obtained by molecular docking.^55–57^ The root-mean-square deviation (RMSD) and root-mean-square fluctuation (RMSF) were used to evaluate the stabilities of the complexes through C-α of the ATIII presented in the Figure 5a–d, respectively. The ATIII-HEP (Figure 5a), ATIII-EX5 (Figure 5b), and ATIII-intact SP-HM (Figure 5c) complexes reached the equilibrium from 60, 50, and 30 ns, respectively. Concerning these time intervals, the ATIII-HEP complex registered the average RMSD values for the three replicates of 2.12, 2.29, and 2.81 Å, while that for the ATIII-EX5 were of 2.50, 2.49, and 2.40 Å. On the other hand, the ATIII-intact SP-HM complex exhibited average RMSD values of 2.24, 2.33, and 3.34 Å for each replicate. RMSF is another useful parameter for analysis that provides information about protein fluctuations and conformational changes.^57^ In Figure 5d, the RMSF results indicated that the HEP-binding domain region that consists of the amino acid residues Lys-114, Lys-125, and Arg-129 ^32^ exhibited low RMSF values concerning the other residues from ATIII, especially in the ATIII-HEP and ATIII-intact SP-HM complexes.

**Figure 5:**
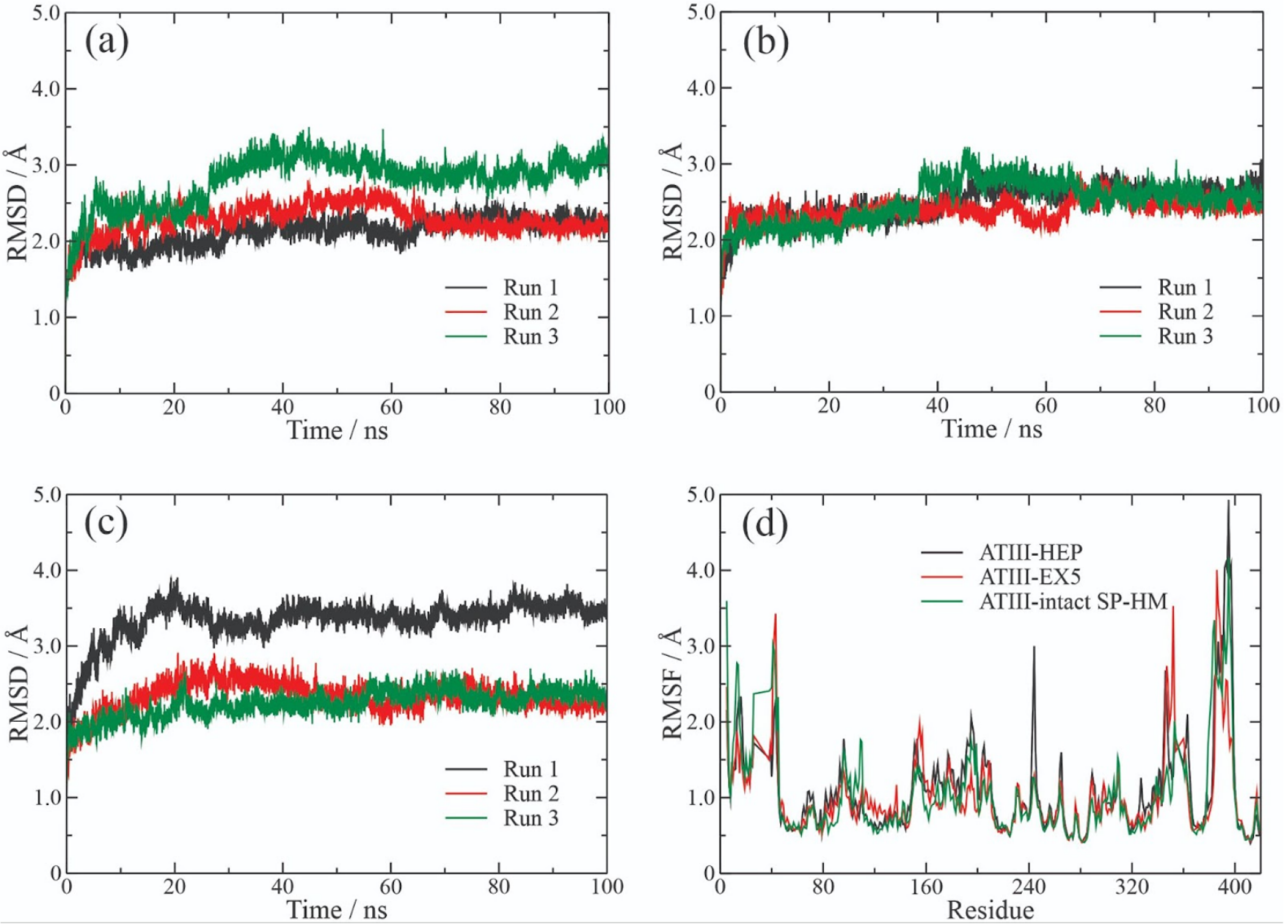
Molecular dynamics stability analyses of ATIII complexes with different ligands. (a–c) Root-mean-square deviation (RMSD) of backbone atoms for each replicate trajectory (black, red, and green lines) of the complexes ATIII–HEP, ATIII–SP-Hm hydrolysate fraction EX5, and ATIII–intact SP from SP-Hm. (d) Root-mean-square fluctuation (RMSF) profiles of ATIII residues when bound to HEP (black), EX5 (red), and intact SP-Hm (green). RMSD and RMSF analyses were used to evaluate the structural stability and residue-level flexibility of the protein–ligand complexes throughout the 100 ns molecular dynamics simulations.

IPE analysis was performed at the interval times when the complexes reached equilibrium, as indicated by the RMSD results (Figure 5a–c). ATIII-HEP, ATIII-EX5, and ATIII-intact SP-Hm complexes registered average IPE values of −1525.91, −1205.23, and −1234.27 kJ mol^–1^, respectively. HEP exhibited the highest interaction potential with the ATIII concerning the EX5 and intact SP-Hm molecules. Analyzing the IPE values for each replicate (Table S1 in the Supporting Information), it was observed that the Coulomb energy is responsible for the main contribution in the IPE values in three replicates in all systems analyzed, indicating that this interaction occurs mainly electrostatically between ATIII and the HEP, EX5, and intact SP-Hm molecules.

Table 4 shows the average energies obtained from MM/PBSA simulations in the last 10 ns for the ATIII-HEP, ATIII-EX5, and ATIII-intact SP-Hm complexes. The energies obtained from these simulations for each replicate for these complexes are presented in the Supporting Information in Tables S2–S4. The ATIII-HEP, ATIII-EX5, and ATIII-intact SP-Hm complexes registered the Δ*G*_bind_ values of −4875.81, −2802.59, and −3454.13 kJ mol^–1^, respectively. The negative Δ*G*_bind_ values indicate that the interaction in all complexes is spontaneous. The main contribution to these values is attributed to the low Δ*E*_ele_ for all systems analyzed, indicating that the HEP, EX5, and intact SP-Hm molecules mainly interact through electrostatic interactions with the ATIII target, as also observed in the IPE results. Furthermore, the Δ*E*_ele_ is also responsible for the strongest interaction observed in the Δ*G*_bind_ values for the ATIII-HEP complex due to the lowest Δ*E*_ele_ values concerning the other complexes. Therefore, the binding energy results indicated that all molecules have spontaneous interactions with the ATIII target, and that the HEP molecule has the strongest interaction with the ATIII target, followed by the intact SP-Hm and EX5 molecules.

**Table 4:** Average Energy Values of the ATIII-HEP, ATIII-EX5, and ATIII-intact SP-Hm Complexes Obtained through MM/PBSA Simulations

energy/kJ mol^–1^	ATIII-HEP	ATIII-EX5	ATIII-intact SP-Hm
Δ*E*_vdW_	–115.01	–322.01	–319.24
Δ*E*_ele_	–7182.68	–3589.21	–4800.13
Δ*G*_polar_	2448.29	1147.12	1708.44
Δ*G*_nonpolar_	–26.41	–38.50	–43.20
Δ*G*_bind_	–4875.81	–2802.59	–3454.13

## 4 Discussion

The present study demonstrates that SPs from the red seaweed *H. musciformis* exhibit promising anticoagulant properties. The significant prolongation of APTT by both the intact polysaccharide (35.85%) and hydrolysate EXP 5 (28%) suggests an effect on the intrinsic and/or common coagulation pathways.^58^ The extraction yield obtained in our study is in line with previous reports for *H. musciformis*, although variations can occur depending on methodology and algal source.^13,43^ In contrast, the lack of substantial alteration in PT indicates a selective mechanism of action, which may be clinically advantageous by reducing interference with the extrinsic pathway.^58^

LMWH enoxaparin (CLEXANE), used as a positive control, significantly prolonged clotting time in the APTT assay at the concentrations tested (0.01 and 0.1 μg mL^–1^). These results have now been included in Figure 4a for direct comparison with SP-Hm and its hydrolysates. It is important to note that, in clinical practice, the anticoagulant activity of enoxaparin is not monitored by APTT but by anti-Factor Xa assays, with recommended therapeutic plasma ranges of 0.5–1.0 IU mL^–1^ for twice-daily administration and 1.0–2.0 IU mL^–1^ for once-daily administration.^59,60^ These values cannot be directly translated into clotting times in global assays such as APTT or PT, since enoxaparin shows limited sensitivity in these tests. In contrast, the SPs from *H. musciformis* selectively prolonged APTT without affecting PT and exhibited a significantly lower hemorrhagic risk *in vivo* compared to enoxaparin. Although no therapeutic range has yet been established for these experimental polysaccharides, the combined *in vitro* and *in vivo* data suggest that SP-Hm may exert anticoagulant effects at concentrations associated with a more favorable safety profile than enoxaparin.

These findings align with previous studies on marine SPs that act via similar anticoagulant mechanisms. Zhang et al. reported that sulfated marine glycans interact with ATIII, a key regulatory protein in the coagulation cascade, supporting the hypothesis that *H. musciformis* exerts its effect through analogous molecular interactions.^61^ Moreover, Wang et al. emphasized that molecular weight and structural integrity are critical to anticoagulant activity, corroborating our results showing that the intact polysaccharide was more effective than its hydrolysates.^62^ Our FT-IR results confirm the characteristic signals of κ-carrageenan, consistent with previously reported spectra for commercial samples.^46^

*In vivo* bleeding time assays further reinforce the therapeutic potential of these polysaccharides, indicating that *H. musciformis* samples may induce lower hemorrhagic risk than LMWH.^63^ This is especially relevant given the well-documented bleeding complications associated with LMWH, despite its effectiveness in thrombosis prevention.^64^ Bleeding time with intact polysaccharide was 1936 s (1.37-fold normal bleeding time), compared to 9633 s with LMWH (6.8-fold the normal bleeding time). These findings indicate that SP-Hm prolonged bleeding time to a much lesser extent than LMWH at the tested concentrations, suggesting a reduced impact on primary hemostasis. While this may point to a potential safety advantage, definitive conclusions require evaluation of thrombosis prevention efficacy and parallel safety assessments at doses producing equivalent anticoagulant effects to LMWH. Similar results were obtained with SP from *G*. *acerosa*, which also demonstrated limited prolongation of bleeding time *in vivo*.^53^

Computational simulations complement these experimental results, clarifying the interactions that occur in the ATIII-HEP, ATIII-EX5, and ATIII-intact SP-Hm complexes. IPE and Δ*G*_bind_ results indicated a strong interaction between the ATIII target and the molecules analyzed, especially with HEP. However, as previously cited, HEP has adverse effects. Then, the second strongest Δ*G*_bind_ value was of ATIII-intact SP-Hm complex, indicating that intact SP-Hm is a promising candidate for use as an antithrombotic agent, as its mechanism is the same as that described for HEP.^58^ It is important to note, however, that the term “strong interaction” refers to the computational prediction of stable and favorable binding between intact SP-Hm and ATIII, and does not imply that its anticoagulant activity should necessarily exceed that of LMWH in biological assays. The anticoagulant response observed *in vitro* and *in vivo* results from a combination of factors—such as molecular weight, sulfation pattern, and pharmacokinetics—and therefore, despite showing lower prolongation of APTT and bleeding time than LMWH, SP-Hm exhibited selective anticoagulant activity with a significantly reduced hemorrhagic tendency. This safety profile may represent an advantage for its potential therapeutic use.

The low binding energy observed for the intact SP-Hm molecules with ATIII highlights the importance of structural features, reinforcing the role of sulfotransferase-mediated modifications as proposed by Meneghetti et al. in defining anticoagulant function through structure–activity relationships.^65^ These results underscore the need for precise structural attributes to ensure optimal biological activity.

Nevertheless, the limitations of the current study must be acknowledged. The predominance of *in vitro* and computational approaches necessitates expansive *in vivo* validation. While initial insights from the bleeding time assay are promising, comprehensive clinical trials are imperative for confirming efficacy and delineating optimal dosing regimens. Prior research has similarly underscored the need for rigorous investigation into the pharmacokinetics and pharmacodynamics of such polysaccharides to fully realize their therapeutic potential in managing thrombotic disorders.^64,66^ Additionally, mechanistic studies, such as evaluating specific factor inhibition (*e.g.*, factor Xa or IIa), are essential to elucidate the exact molecular targets within the coagulation cascade. These steps will be crucial to advancing the clinical development of marine-derived SPs as novel anticoagulant agents.

The hydrolysis design successfully generated sulfated galactans of different molecular weights, and there are indications that the antithrombotic and anticoagulant activities are related to molecular weight. The intact SP-Hm and the hydrolysates act in the coagulation cascade’s intrinsic and/or common pathway, not the extrinsic pathway. The results of the *in silico* analyses indicate that the mechanism of action of PS-Hm is similar to that of HEP, specifically in activating ATIII, an inhibitor of the coagulation cascade. Furthermore, it can be concluded that ATIII interacts more strongly with higher molecular weight PS-Hm compared to its hydrolysate, corroborating the *in vitro* assays. Additionally, the reduction in molecular weight also decreased the hemorrhagic tendency. This observation agrees with previous findings showing that intact carrageenans exhibit stronger anticoagulant activity compared to their low–molecular weight derivatives.^48^

## 5 Conclusions

This study demonstrated that SPs extracted from *H. musciformis*, particularly the intact polysaccharide and the 231 kDa hydrolysate (EX5), exhibit selective anticoagulant activity on the intrinsic coagulation pathway, as evidenced by a significant prolongation of APTT without affecting PT. *In vivo*, these samples did not significantly increase bleeding time compared to LMWH, indicating a more favorable safety profile.

Our computational analyses confirmed that the interaction between intact SP-Hm and ATIII is stable and spontaneous, suggesting a mechanism similar to that of HEP. Experimental and computational results indicate *H. musciformis* SPs have good anticoagulant efficacy with low hemorrhagic risk. This offers a safer and more sustainable alternative to animal-based HEPs.

This is the first integrated evidence from *in vivo*, *in vitro* and *in silico* studies that carrageenan-type polysaccharides of marine origin can act as anticoagulants and with a low risk of bleeding. The observed molecular weight-dependent activity highlights opportunities for structure–activity optimization to balance efficacy and safety.

Future studies should include thrombotic disease models and pharmacokinetic/pharmacodynamic analyses to define effective dosing and evaluate clinical potential. Overall, *H. musciformis* polysaccharides emerge as promising candidates for next-generation nonanimal anticoagulant therapies.

## Supplementary Material



## Notes

Energies and interaction parameters for ATIII–ligand complexes: Coulomb, van der Waals, and IPE values for ATIII–HEP, ATIII–EX5, and ATIII–intact SP-HM complexes (Table S1); MM/PBSA energy components for ATIII–HEP complex (Table S2); MM/PBSA energy components for ATIII–EX5 complex (Table S3); MM/PBSA energy components for ATIII–intact SP-HM complex (Table S4) (PDF)
