# Supplementary material for: The Antithrombotic Potential of Sulfated-Polysaccharides from Red Seaweed Hypnea musciformis (Wulfen) J.V. Lamouroux: An In Vitro, In Silico and In Vivo Study
Source: ACS Omega. 2026 Jan 16;11(4):5181–92. doi: 10.1021/acsomega.5c07355 (PMC12878786; doi:10.1021/acsomega.5c07355)
Supplement: Supplementary file 1 [file ao5c07355_si_001.pdf]

SUPPLEMENTARY MATERIAL

The antithrombotic potential of sulfated-polysaccharides from red seaweed *Hypnea musciformis* (Wulfen) J.V. Lamouroux: an *in vitro*, *in silico* and *in vivo* study

Caroline L. Peixoto[1], Vitória Karoline F. Monteiro[1], José Osmar S. Júnior[3], Lucas L. Bezerra[3], George Meredite C. de Castro[4], Norberto de Kássio V. Monteiro[3*], Renato de Azevedo Moreira[4], Aline M. A. Martins[5], Ludmila Belayev[2], Reinaldo B. Oriá[1]

1. Laboratory of the Biology of Tissue Healing, Ontogeny and Nutrition, Department of Morphology and Institute of Biomedicine, School of Medicine, Federal University of Ceara, Fortaleza-CE, Brazil
2. Neuroscience Center of Excellence, School of Medicine, Louisiana State University Health Sciences Center, New Orleans, LA, USA.
3. Department of Analytical Chemistry and Physical Chemistry, Federal University of Ceara, Fortaleza-CE, Brazil.
4. Department of Biochemistry and Molecular Biology, Federal University of Ceara, Fortaleza-CE, Brazil.
5. Integrated Space Stem Cell Orbital Research (ISSCOR) Center / Sanford Consortium for Regenerative Medicine - UCSD. Center for Novel Therapeutics - 9310 Athena Cir, suite 200, La Jolla, CA 92037.

Table S1 - Coulomb energy, van der Waals energy, and IPE values with standard deviation for each replicate of the ATIII-HEP, ATIII-EX5, and ATIII-intact SP-HM complexes.

Systems	Replicate	Coulomb energy / kJ mol^{-1}	van der Waals energy / kJ mol^{-1}	IPE / kJ mol^{-1}
ATIII-HEP	1	-1410.33 (±152.41)	-100.39 (±29.53)	-1510.72 (±153.45)
	2	-1525.01 (±142.66)	-95.03 (±27.98)	-1620.04 (±138.06)
	3	-1359.50 (±144.44)	-87.48 (±25.48)	-1446.98 (±141.08)
ATIII-EX5	1	-857.09 (±105.43)	-320.64 (±37.12)	-1177.73 (±124.64)
	2	-953.96 (±126.09)	-292.45 (±30.67)	-1246.41 (±155.28)
	3	-918.64 (±95.63)	-272.91 (±32.40)	-1191.55 (±168.71)
ATIII-intact SP-HM	1	-925.34 (±156.69)	-218.85 (±43.62)	-1144.19 (±183.81)
	2	-885.32 (±154.63)	-324.26 (±51.77)	-1209.58 (±144.76)
	3	-1132.51 (±142.62)	-216.52 (±33.94)	-1349.03 (±139.37)

Source: author

Table S2 - Energies values for each replicate of the ATIII-HEP complex obtained through MM/PBSA simulations.

Energy / kJ mol^{-1}	Replicate 1	Replicate 2	Replicate 3
ΔE_{vdW}	-110.75	-128.12	-106.16
	(±33.17)	(±27.52)	(±25.40)
ΔE_{elect}	-6843.64	-7996.17	-6708.23
	(±382.76)	(±372.52)	(±300.13)
ΔG_{polar}	2295.78	2789.21	2259.87
	(±184.42)	(±156.75)	(±108.68)
$\Delta G_{non\text{-}polar}$	-25.38	-30.26	-23.58
	(±3.07)	(±2.21)	(±1.78)
ΔG_{bind}	-4683.99	-5365.34	-4578.10
	(±237.23)	(±239.94)	(±233.46)

Source: author

Table S3 - Energies values for each replicate of the ATIII-EX5 complex obtained through MM/PBSA simulations.

Energy / kJ mol^{-1}	Replicate 1	Replicate 2	Replicate 3
ΔE_{vdW}	-303.96	-340.06	-321.99
	(±21.80)	(±25.51)	(±34.55)
ΔE_{elect}	-3461.88	-3749.34	-3556.40
	(±211.27)	(±169.25)	(±294.60)
ΔG_{polar}	1005.60	1326.33	1109.43
	(±116.95)	(±74.27)	(±168.51)
$\Delta G_{non\text{-}polar}$	-34.47	-41.61	-39.42
	(±2.51)	(±2.02)	(±4.82)
ΔG_{bind}	-2794.71	-2804.68	-2808.38
	(±141.31)	(±142.78)	(±176.76)

Source: author

Table S4 - Energies values for each replicate of the ATIII-intact SP-HM complex obtained through MM/PBSA simulations.

Energy / kJ mol^{-1}	Replicate 1	Replicate 2	Replicate 3
ΔE_{vdW}	-320.85	-391.87	-245.01
	(±33.25)	(±31.71)	(±31.50)
ΔE_{elect}	-4507.89	-4526.56	-5365.94
	(±251.86)	(±339.09)	(±236.25)
ΔG_{polar}	1669.07	1566.01	1890.23
	(±151.98)	(±172.96)	(±118.96)
$\Delta G_{non-polar}$	-45.68	-47.78	-36.13
	(±3.10)	(±3.01)	(±2.86)
ΔG_{bind}	-3205.35	-3400.20	-3756.85
	(±177.66)	(±213.29)	(±171.99)

Source: author
